# Are There More Human Cancer Viruses Left to Be Found?

**DOI:** 10.1146/annurev-virology-111821-103721

**Published:** 2024-09

**Authors:** Patrick S. Moore, Yuan Chang

**Affiliations:** Cancer Virology Program, Hillman Cancer Center, University of Pittsburgh, Pittsburgh, Pennsylvania, USA

**Keywords:** virus discovery, cancer virology, human tumor viruses, Kaposi sarcoma herpesvirus, KSHV, Merkel cell polyomavirus, MCV

## Abstract

Of the thousands of viruses infecting humans, only seven cause cancer in the general population. Tumor sequencing is now a common cancer medicine procedure, and so it seems likely that more human cancer viruses already would have been found if they exist. Here, we review cancer characteristics that can inform a dedicated search for new cancer viruses, focusing on Kaposi sarcoma herpesvirus and Merkel cell polyomavirus as the most recent examples of successful genomic and transcriptomic searches. We emphasize the importance of epidemiology in determining which cancers to examine and describe approaches to virus discovery. Barriers to virus discovery, such as novel genomes and viral suppression of messenger RNA expression, may exist that prevent virus discovery using existing approaches. Optimally virus hunting should be performed in such a way that if no virus is found, the tumor can be reasonably excluded from having an infectious etiology and new information about the biology of the tumor can be found.

## POSING A PROVOCATIVE QUESTION

Hepatitis C virus (HCV) causes about one-third of liver cancers worldwide, and its discovery led to the 2020 Nobel Prize awarded to Houghton, Alter, and Rice. A good place to look for cancer viruses like HCV might be The Cancer Genome Atlas (TCGA) project, a highly annotated and curated database of exhaustively sequenced cancer tissue messenger RNAs (mRNAs). Various groups used TCGA databases to search for new cancer viruses, with none being found, and this seems to be strong evidence against finding more human cancer viruses (1). However, RNA from HCV, a known and frequent cause of hepatocarcinoma, has not been found in TCGA (2). This positive-stranded RNA virus is not polyadenylated, may persist in circularized forms (3), and thus cannot be identified for technical reasons in most TCGA sequencing projects—although, ironically, contaminating HeLa human papillomavirus 18 (HPV18) and xenotropic murine leukemia virus sequences can be found (2, 4). There is every reason to suppose that the next human tumor virus may have an unforeseen quirk like HCV.

The Editorial Committee of the *Annual Review of Virology* asked us to address the question, “Are there more human cancer viruses left to be found?” This is not trivial because viruses are known to cause at least 15% of all human cancer cases (5), yet they often remain overlooked by clinicians, vaccine makers, pharmaceutical companies, and government health policy experts. We cannot give a definitive answer to this question, and so we will try to frame it within the context of our experience with two different cancer viruses. But the question itself is also loaded, with almost each word being a scientific landmine. The answer depends on the meanings of the words “virus,” “cause,” and “cancer.”

Human viral cancers are currently attributable to only seven viruses that are maintained in the human population and cause cancers (Table 1). But additional viruses should be noted as well. Human immunodeficiency virus (HIV) does not generally initiate cancer on its own, although rare monoclonal proviral insertions in T cell and macrophage tumors have been reported (6). Persons with AIDS (acquired immunodeficiency syndrome), however, have a markedly increased risk of select cancers compared to their reference populations due to immune suppression (7, 8). These cancers are most often caused by coinfection with one or more of the other established human tumor viruses. This relationship is strong enough that HIV infection together with Kaposi sarcoma (KS), cervical cancer, or Burkitt lymphoma is sufficient to meet a Centers for Disease Control and Prevention (CDC) case definition of AIDS, regardless of the patient’s CD4^+^ cell count (9). For these reasons, HIV is also considered to be a carcinogen (10). In addition, insertional mutagenesis and T cell leukemia have been reported as consequences of retroviral vector gene (11) and chimeric antigen receptor T cell therapies (12). Another example of iatrogenic viral tumorigenesis involves the BK polyomavirus (BKV) for which there are convincing case reports of clonally integrated virus in some urothelial cancers after patients have undergone kidney transplantation (13). So far, there is little evidence for BKV playing a broader role in genitourinary tumors, but this remains a possibility (14). These examples suggest that additional known viruses might be found to contribute to specific human cancers, such as the recent discovery of HPV42 in digital papillary adenocarcinomas (15).

Returning to the seven main culprits, the most striking feature about these viruses is not what they have in common but rather their differences. They represent most of the different Baltimore virus classification groups, and commonalities among them are elusive (16). All seven have close viral relatives that do not cause cancer. This list, in fact, suggests that almost any virus has the potential to cause cancer, but only a very few viruses actually do. Two hepatitis viruses, hepatitis B virus (HBV) and HCV, cause nearly indistinguishable liver cancers but have completely different biologies and no genes in common. We have described some of the general characteristics of human tumor viruses elsewhere (17) and will only briefly touch on these features here.

## CANCER FORMATION BY VIRUSES IS A BIOLOGICAL ACCIDENT

One tenet from studies of both human and animal cancer viruses is that cancer formation in the natural host is a biological accident since it confers no evolutionary benefit to the virus (18). Viral transmission, the sole determinant for viral evolutionary fitness, is usually asymptomatic for the tumor viruses, and tumors emerge only in a minority of infected persons. Epstein-Barr virus (EBV), for example, is a near-universal infection of adults (>95%), and the vast majority of EBV transmission events cause no symptomatic disease—not even infectious mononucleosis, let alone EBV-related malignancy. When tumors do occur, the virus is largely nontransmissible and tumor formation is not part of the virus’s life cycle. Epstein, Achong, and Barr (19) discovered encapsidated herpesvirus virions in Burkitt lymphoma by electron microscopy in 1964, but only rare tumor cells, possibly triggered by a delayed receipt of the sample for analysis samples, were responsible for generating the virions that were found. Most of these rare virion-generating tumor cells were likely destined to die from virus production.

Active viral replication and capsid formation (so-called lytic replication) cause a cytopathic effect (CPE), killing the infected host cell. CPE is not specific to any class of viruses or cells; it is a stereotypic cellular innate immune response to virus replication. Hence, plaque assays can be used to quantitate many different RNA, DNA, and retroviruses using a wide variety of cells. Cell death through CPE, of course, is incompatible with tumorigenesis. Despite viral tumors being abundantly infected, they harbor viruses that are in a nontransmissible form that does not provide a survival benefit to the virus.

This raises an important issue: If viruses did not evolve to cause tumors, why do they encode oncoproteins? A long-standing answer has been that viruses initiate unscheduled cell division to promote their own replication, which can lead to cancer cell transformation if specific viral mutations arise (20). This explanation is not obsolete, but it is incomplete. Could it be that scientists have too narrowly classified cellular tumor suppressor proteins (such as p53, etc.) targeted by viral oncoproteins simply because of how these genes were originally discovered?

While these pathways undoubtedly control nonviral cancer cell growth, it has become clear that they also have a broader innate immune surveillance function. Cancer cell growth and viral infection share similar molecular patterns such as cytoplasmic DNA, unprotected DNA ends, and unscheduled cell cycle entry leading to genomic instability (18). It is likely that metazoan tumor suppressor pathways arose from ancestral unicellular innate immune pathways but now defend against both tumor formation and viral infection (21). Nucleic acid sensors, for example, can activate common cellular pathways (e.g., pRB1, p53, and RIG-I signaling pathways) that are operative in host defense against both infection and cancer (22, 23). For prolonged virus survival in the hostile environment of a cell, blunting of these shared innate immune/tumor suppressor responses appears to be necessary to prevent virus-induced cell cycle arrest, programmed host cell death, and activation of adaptive immune responses—the same cellular responses that prevent the emergence of cancers (24, 25). The dualism between tumor suppressor and innate immune pathways helps to explain why tumor viruses, as well as viruses that do not cause tumors (25), encode so-called oncogenes.

This principle was first noted for Kaposi sarcoma herpesvirus (KSHV) because of its extensive molecular piracy of host genes involved in immune evasion (25, 26) and then extended to other viruses (22, 27, 28). If the viral life cycle is aborted by mutation in the virus or if the host is unable to mount an effective immune response to control latent viral infection, then there is danger for cancer to arise. It is remarkable that only seven viruses, and only under highly specific conditions, promote tumorigenesis in humans, a property that is not shared even by other closely related viruses (18).

## VIRUSES ASSOCIATED WITH HUMAN TUMORS ARE PERSISTENT INFECTIONS

Another feature of the tumor viruses is that they all cause prolonged human infections. This can be achieved through viral latency, which is an immune evasion mechanism in which most viral genes are suppressed. Unlike an acute viral infection, the latent viral genome does not replicate independently from the host cell genome. Viruses without a recognized latency program, such as HPV18, may require rare mutation and integration events for the viral infection to become carcinogenic (29). If the virus infects a nondividing cell, latency can occur by the virus simply shutting off its own replication machinery as occurs with herpes simplex virus in a G_0_ neuron. A more elaborate mechanism, however, is required for latent viral infections of dividing cells such as with KSHV and EBV in B cell reservoir populations. This can trigger host defense pathways, e.g., cGAS-STING signaling, that require viral inhibition for the latent viral genome to persist (30, 31). Surprisingly, latency is only formally defined for herpesviruses and retroviruses, but some form of latent life cycle may exist for most persistent viruses (32). This is based on a simple exercise in logic: For a nonlatent virus to persist for months or years through continuous productive virus replication, it must replicate perfectly in tandem with its host cell reservoir; otherwise, the virus will either be lost from the host over time, especially through immune surveillance, or will eventually overwhelm the host.

Chronic infection, regardless of how the tumor virus persists, may be required to allow multiple genetic and immunological risk factors to accumulate, each of which is uncommon, that convert a noncarcinogenic infection into a cancer. For this reason, chronic viral infections seem more likely to be candidate carcinogens than acute viral infections.

## ANSWERING A PROVOCATIVE QUESTION

### Human Virus

Getting back to the difficult words in the Editorial Committee’s question, first, what is a human virus? An autonomous, parasitic nucleic acid encoding its own mechanism for transmission between individuals, and thus maintaining itself in the human population, would meet the general definition. But could a prion cause cancer? We do not think so based on known biology, but we would be foolish to completely rule it out.

What about bacteriophages? These are not considered human viruses, but a bad actor could certainly be carried within us through our bacterial flora. We have shotgun sequenced RNA from nonsterile site tumors, such as conjunctival carcinomas, and found enormous numbers of bacteriophage-coding sequences. Is it possible that a bacteriophage might jump into a eukaryotic cell and cause havoc? Perhaps, and this scenario is certainly more biologically plausible than the cancer prion possibility. Moreover, bacteriophages would be maintained in the human population not through their own accord but by piggybacking onto the bacterial host. The application of metagenomics to virus hunting, including long sequencing and tailored bioinformatic analyses, has accelerated the identification of entirely new species and families of viruses, such as giant viruses as well as new RNA and DNA viruses (33–36). The biology of these new agents is not well studied, and they represent previously unidentified dark matter in our considerations of viral carcinogenesis.

The edges of possibility for a human cancer virus become even more tenuous when considering endogenous human viruses including endogenous retroviruses (ERVs) that were presumably once exogenous but became endogenized during human evolution. Some viruses, such as endogenous human herpesvirus 6, are even integrated at sites such as telomeres, providing a ready-made molecular cancer mechanism (37).

A most interesting and vexing example of cancer caused by a retrovirus goes all the way back to the beginnings of tumor virology with Peyton Rous’s discovery of Rous sarcoma virus (RSV) that encodes a copy of the chicken *Src* oncogene (38, 39). Similar acutely transforming retroviruses—often from chickens but also from mice, rats, and other animals—led to the discovery of more than 20 different cellular oncogenes captured by these viruses that formed much of our understanding of the basis for the molecular biology of cancer (40).

But Rous’s discovery also may have been responsible for a very bumpy road in tumor virus research (41). Rous performed a unique experiment by first passaging a sarcoma tumor through transplantation into related chickens (38, 42). During serial transplantation passages, the tumor became more aggressive. It was minced and filtered, and the tumor homogenate was reinjected into another chicken from the same brood, causing a new sarcoma tumor (43). These experiments were meticulous and the conclusions obvious, but there are problems in interpreting this discovery. Sarcomas caused by RSV in bird populations are nonexistent, whereas the parental virus, avian leukosis virus (ALV) without the *Src* insertion, is a common chicken pathogen. In fact, the *Src* insertion makes RSV replication defective, and so coinfecting ALV is required as a helper virus for RSV transmission in birds (44, 45). Although tumorigenesis is reproducible after injecting RSV, it does not seem to explain natural avian cancers. By the 1970s, the paradoxes and inconsistencies of natural infections by these viruses, as well as overly enthusiastic descriptions of other ERV tumor viruses [the so-called human rumor viruses (46)], led to tumor virology often being dismissed as a respectable discipline in human oncology (41).

Is it possible that Rous’s discovery was perhaps one of the most fruitful scientific mistakes ever made, leading to a Nobel Prize in 1966? What if Rous’s first chicken tumor was not initially caused by RSV? What if, instead, the original tumor was caused by an amplified or overexpressed cellular *Src* gene that became integrated into a noncarcinogenic ALV, forming RSV (47)? Loss of ERVs and transposon restriction in tumors is well established, and it is not difficult to imagine a retroviral recombination event occurring in a noninfectious tumor (48–50). If this were the case, then Rous’s experimental manipulations (e.g., intramuscular injection) might have allowed this Frankenstein virus, together with its helper virus, an opportunity to infect and cause cancer in chickens that would not have occurred naturally. So which came first, the tumor (chicken) or the virus (egg)? We have no way of knowing, but if RSV was not the original cause of the chicken sarcoma studied by Rous, this helps to explain much of the confusing natural history of RSV and other simple, acutely transforming retroviruses.

If an exogenized, infectious ERV can cause human cancer, searching for it by TCGA would be very difficult because the agent’s sequences would match endogenous human retroviral sequences making up ~5% of our natural genome (51). One could even imagine that genetic transmission of such a virus could lead to immune tolerance so the cancers from such a virus would not have the immune-related risk factors typically seen for viral cancers.

### Cause

Causation is a tricky subject. Most people firmly feel that they know causality when they see it, but even a cursory discussion of cancer causation can provoke controversy. If EBV causes Burkitt lymphomas, why don’t we all have this tumor since nearly everyone harbors EBV infection? More perplexing is the fact that only some Burkitt lymphomas are associated with EBV infection, particularly those occurring in Africa and Papua New Guinea, whereas less than half of sporadic Burkitt lymphomas in the United States are EBV-positive. There are only a handful of cancers—KS and primary effusion lymphoma (PEL) (KSHV), adult T cell leukemia [human T-lymphotropic virus 1 (HTLV-1)], X-linked lymphoproliferative disorders (EBV), and cervical cancer (HPV)—that are universally associated with specific viral infections. For the remaining viral tumors, virus infection usually causes only a fraction of the total tumor cases. These uninfected tumors may result from multiple somatic mutations that phenocopy a viral infection (52). Further, cMyc rearrangements, a somatic mutation, are a hallmark for Burkitt lymphoma, indicating that EBV infection alone is not responsible for this tumor. It would take over 30 years from EBV’s discovery for the International Agency for Research on Cancer to declare that EBV is a carcinogen, and this was based on data from X-linked lymphoproliferative disorders rather than Burkitt lymphoma (53).

Causality is a subjective determination, and there are many tests for it. One test frequently quoted is that a virus must be both necessary and sufficient for a causal relationship to be established. Most disease syndromes, however, have multiple causes, negating the necessity clause as described above for Burkitt lymphoma and EBV. Few would question that pneumonia is caused by multiple agents and, similarly, the existence of multiple causes for a given cancer type can be reasonably accepted. This does not mean that a virus cannot cause some fraction of a cancer type, just as pneumococci cause some but not all cases of pneumonia. Further, every infectious disease—not just cancer—is influenced by noninfectious factors (e.g., preexistent immunity, age, immune competence), and so infection alone is never sufficient. The necessary and sufficient causality test is not useful and is not used in modern epidemiology.

What about Koch’s postulates? These were formulated in 1884, but viruses cannot be “isolated in pure culture,” so these are no more helpful than the necessary/sufficient test (54). In one remarkable example, a suspected new human hepatitis virus was actually found to be a contaminant virus from the diatomaceous earth used in RNA isolation columns, revealing how difficult it is to achieve a pure virus culture (55). Furthermore, given the species specificity of viruses, humans are often the only susceptible host. Therefore, Koch’s postulates (requiring passage and recapitulation of disease in another host) also cannot be rigorously used to evaluate cancer causation and have little value to human cancer virology.

The most common causality test used by epidemiologists is the list of criteria developed by A.B. Hill (56) to assess a causal relationship between cigarette smoking and lung cancer. These criteria, while valuable, also have shortcomings for cancer virology (57). Some virus-cancer relationships such as KSHV and KS (58) are well behaved using Hill’s criteria, while others (EBV and Burkitt lymphoma) are not.

There is one cancer-virus mechanism that is difficult to address, the so-called hit-and-run mechanism, where a causal viral infection could initiate tumorigenesis through cellular mutations and then is lost as the tumor matures. We are unaware of any currently known cancer virus that does this. Viral hepatocellular carcinoma occurs after chronic, decades-long infection that first usually generates cirrhosis and then, ultimately, hepatocellular carcinoma. Not only is the viral cause due to a chronic infection, not a hit-and-run event, but also for HBV, the genome is generally clonally integrated into the hepatocellular carcinoma cells (59, 60). HCV, a cytoplasmic, positive-stranded RNA virus that is not thought to be capable of directly promoting oncogenesis, is still generally persistent in persons developing HCV-related liver cancer (61). Neither of these infections meet the pattern expected for a hit-and-run mechanism. The remaining five viruses in Table 1 carry genes required for cancer cell growth and are readily detected in their tumors (62, 63). But if hit-and-run does occur, supporting evidence can come from well-designed seroepidemiology studies [as has been done, for example, with EBV and multiple sclerosis, a non-neoplastic autoimmune disorder (64)]. In our view, a hit-and-run mechanism should be a hypothesis of last resort and made only with the strongest supporting evidence.

Accurate genome quantitation is critical to assessing causality since, at least for directly transforming viruses, each cancer cell possesses at least one viral genome. Future causation might be best determined through quantitative Bayesian inference of rigorous experiments to determine whether a virus-tumor association is likely to be coincidental or causal. A noncausal relationship between a virus and a cancer can be excluded when a rigorously designed quantitative study generates a posterior probability such that a noncausal relationship can be essentially ruled out. In the example of EBV-positive Burkitt lymphoma, EBER1 RNA can be detected in virtually all tumor cells in the EBV-associated tumors and the EBV virus in these tumors is clonal (65). Coincidental EBV infection occurs in healthy persons, in contrast, at a level of ~1 in 100,000 peripheral mononuclear blood cells [i.e., mainly B cells (66)] and is nonclonal or oligoclonal. Based on these findings alone, the probability of coincidental EBV infection in these tumors is extremely low. The high prevalence of EBV infection precludes assessment by standard epidemiological methods such as Hill’s criteria, but these molecular findings provide strong quantitative evidence for a causal relationship in EBV-positive Burkitt lymphomas.

This may not be as satisfying as an epidemiological checklist, but this type of Bayesian reasoning may be needed and may become possible with future molecular biology technologies. This was informally done by the scientific community for Merkel cell polyomavirus (MCV or MCPyV) and Merkel cell carcinoma (MCC), in which there is no serious doubt about a causal relationship. MCV, like EBV and Burkitt lymphoma, is a near-ubiquitous human infection that causes a large fraction of MCC tumors. In virus-positive MCC, MCV is clonally integrated and mutated in the tumor cells and it is not replication competent (67, 68). This indicates that the virus was present prior to cancer cell formation, which makes the noncausal hypothesis exceedingly unlikely.

As we learn more about the molecular nature of viruses and of tumors, our definitions of causality may change and become more nuanced. Philosopher of science Karl Popper described scientific conclusions, such as causality, by analogy to a structure built on piles in a swamp: “If we stop driving the piles deeper, it is not because we have reached firm ground…we are [just] satisfied that the piles are firm enough to carry the structure, at least for the time being” (69).

### Cancer

**Figure 1:**
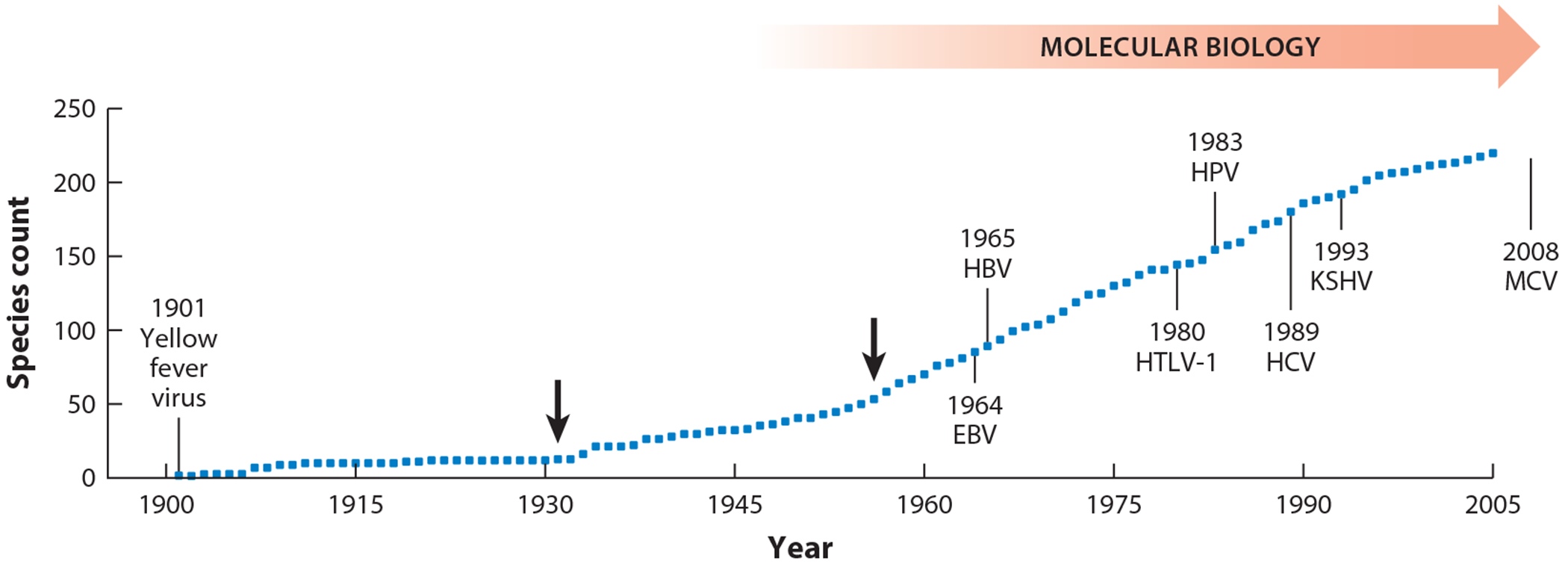
Virus discovery curve showing cumulative number of species reported to infect humans up to 2005. Statistically significant upward breakpoints in the discovery curve are indicated with arrows. All known tumor viruses were discovered in the latter part of the last century, coinciding in part with advances in molecular biology. Abbreviations: EBV, Epstein-Barr virus; HBV, hepatitis B virus; HCV, hepatitis C virus; HPV, human papillomavirus; HTLV-1, human T-lymphotropic virus 1; KSHV, Kaposi sarcoma herpesvirus; MCV, Merkel cell polyomavirus. Figure adapted from Reference 130 (CC BY 3.0).

The development, accessibility, and sophistication of molecular cancer biology have been driving factors in new pathogen discovery (Figure 1). But the cancers that have been exhaustively sequenced are almost exclusively from North American and European populations. Some cancers that have been so far ignored are critically important to low- and middle-income countries (LMICs) in which viruses are known to have a predominant role. KS occurring in a person with HIV, for example, is by definition a case of AIDS, and unfortunately AIDS patients are commonly not counted in cancer registries. One study of global cancer trends ignored KS precisely for this reason (70) despite both AIDS-KS and HIV-negative KS being some of the most frequent cancers in many sub-Saharan African countries. It is uncertain how much undercounting for KS occurs in global World Health Organization cancer surveillance [Global Cancer Observatory (GCO)], but it may be as high as ~80–90% based on the known occurrence rate of AIDS-KS among AIDS patients. Other unusual cancers suspected to have an infectious etiology in LMICs include conjunctival carcinoma and some forms of esophageal carcinoma. While it is possible that all viruses causing cancer in wealthier countries have been found, it is certain that cancers in LMICs have not had this same level of molecular biology scrutiny.

## KAPOSI SARCOMA HERPESVIRUS

Our experience in isolating two new DNA tumor viruses, KSHV (also called human herpesvirus 8 or HHV8) and MCV, may be useful to illustrate some of these issues in cancer virus discovery. There are excellent reviews that describe the discoveries of each of the other cancer viruses: EBV (71), HBV (72), HTLV-1 and HIV (73), HPV (74), and HCV (75). We urge the reader to explore these accounts as well to gain a more balanced view of cancer virus discovery.

KS was initially described by Moritz Kaposi in 1872 (76) and in subsequent decades remained an enigmatic tumor. While KS was generally rare, when it did occur, it tended to occur in elderly persons, particularly males, having Mediterranean or Ashkenazi Jewish ethnicity. By the mid-1970s, however, KS emerged as one of the more common tumors in early organ transplant recipients, illustrating the importance of cellular immunity to this cancer (77). Also, at about the same time, colonial and postcolonial medical surveys conducted prior to AIDS (often performed by Denis Burkitt) determined KS to be frequently seen in parts of eastern and southern Africa, and it was the third or fourth most common cancer in some countries (78). The emergence of AIDS as a recognized syndrome was concurrent with the epidemic of KS, particularly among young, previously healthy gay and bisexual men in the early 1980s (79). Hypotheses on how this could occur ranged from the obvious (an unknown viral infection) to the bizarre (homosexual activity causing immune suppression through some form of immunologic hyperstimulation). Some suspected that KS might be a direct manifestation of HIV infection, particularly via expression of the HIV tat protein. Others felt KS was an inflammatory cytokine-induced hyperplasia. Various viruses and microbes also were suggested to be the cause including cytomegalovirus (CMV) (80), papillomaviruses (81), and mycoplasma (82).

Epidemiology rather than molecular virology was key to clarifying this very confusing scientific picture. In a classic paper, Beral, Peterman, Berkelman, and Jaffe used CDC data to define a likely agent (83):

1. KS is not unique to AIDS, but the rate is increased up to twenty-thousand-fold by HIV immune suppression.
2. KS is likely caused by an uncommon agent in most European and North American populations, but the agent’s prevalence is increased in Mediterranean populations and high in sub-Saharan African populations.
3. HIV is not the direct cause of KS since KS is rare among transfusion recipients with AIDS. Gay/bisexual men with AIDS were found to have a 20-fold higher risk for KS than male AIDS patients from other HIV-transmission risk groups, despite both groups being similarly HIV-positive and immunosuppressed, suggesting that the cancer is caused by an entirely different sexually transmitted infection.

These features were inconsistent with any then-known infectious agents.

**Figure 2:**
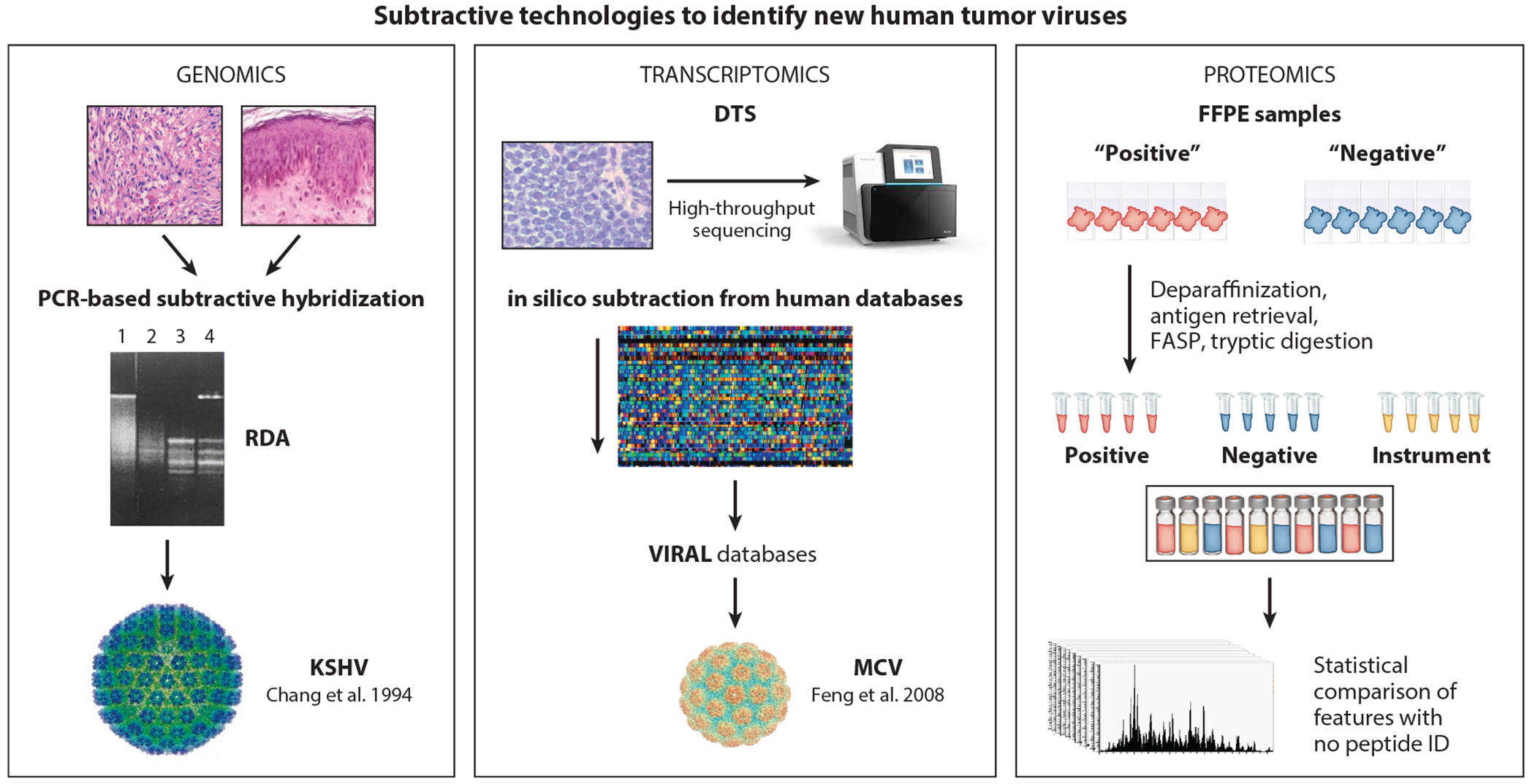
Subtractive technologies to identify new human tumor viruses. Cancer virus discovery technologies have evolved from differential detection of cancer viral DNAs using RDA (84) (*left*) to identifying unique viral messenger RNAs using transcriptomic subtraction (67, 113) (*middle*) to sequencing unique viral peptides that can be used to infer viral nucleotide sequences (128) (*right*). The critical problem solved in each case relies on subtraction of human sequences in tumor samples to obtain unique viral sequences. Abbreviations: DTS, digital transcriptome subtraction; FASP, filter-aided sample preparation; FFPE, formalin-fixed paraffin-embedded; KSHV, Kaposi sarcoma herpesvirus; MCV, Merkel cell polyomavirus; RDA, representational difference analysis.

In 1993, Lisitsyn, Lisitsyn, and Wigler (84) published a description of a competitive subtractive PCR approach to physically identify differences between two DNA genomes, called representational difference analysis (RDA) (Figure 2). This method employed isolating two DNA genome samples and cutting them both to completion with restriction enzymes. PCR linkers were then ligated to the restriction sites on DNA only in the sample predicted to have an undiscovered virus [RDA could also be used to identify mutations if a different restriction pattern is caused by the mutation and was later used to co-discover the PTEN tumor suppressor gene (85)]. Melting and hybridizing this DNA with an excess of the unligated, digested healthy tissue DNA were followed by PCR using primers recognizing the ligated linker. If a unique DNA fragment is present only in the diseased tissue DNA, the fragment would rehybridize to itself with adapter primers on both strands and would be exponentially PCR amplified. Genomic fragments present in both diseased and healthy tissue genomes would reanneal to each other and, with the healthy tissue DNA in excess, would have only one primer to arithmetically amplify. Iterative rounds of PCR and hybridization would tend to enrich DNA fragments present only in the diseased sample. The potential for success with RDA in identifying a foreign sequence in a human tumor genome was based on the insight that simplifying the complexity of the six billion base pair genome by selectively using amplifiable DNA restriction fragments between 300 and 1,000 base pairs in size would be essential.

We started collaborating to determine if viruses of public health concern could be discovered using molecular biology techniques rather than by traditional methods such as electron microscopy or virus culture. Based on the Beral et al. paper (83), we chose to search for an agent using RDA on DNA isolated from tissues from an AIDS-KS autopsy case. The HIV epidemic by then had approached its peak in the United States, and no effective treatment or prevention was available. Entire wards of New York hospitals were turned over to near-hospice care of AIDS patients, many with KS. A patient’s KS tumor and healthy control tissues to be used in RDA were dissected under sterile conditions to avoid false-positive results from flora DNAs. Ironically, this case was one of the last AIDS-KS patients to undergo an autopsy at Columbia University for the next several years because HIV transmission risk to morgue personnel was deemed too high.

RDA generated four DNA fragments that were cloned and sequenced for use as Southern and PCR probes (86). Two RDA bands appeared to be human sequences and were not investigated further. The other two BamHI fragments (330 and 631 bases in length termed KS330Bam and KS631Bam, respectively) were specific to KS and not healthy tissue. Unfortunately, routine sequence analysis in 1993 was still too crude to determine if these bands belonged to a new virus. We were fortunate to work with Ethel Cesarman and Dan Knowles, colleagues in hematopathology, who had a large collection of AIDS and AIDS-KS tissues that could be examined. All 25 KS tumors with intact DNAs that were tested were positive for the RDA bands, while 49 various surgical biopsy tissue DNAs from patients without KS or HIV infection were uniformly negative, confirming their specificity to KS tumors.

KS tumors from non-AIDS patients from the United States and both HIV-positive and -negative patients from Africa were also positive for KS330Bam and KS631Bam (87, 88). Thus, the association of RDA-derived DNA fragments seemed both generalizable and specific to KS, fulfilling several of Hill’s criteria for causality. Independent confirmation of these results was obtained by blinded testing of samples by Robin Weiss’s group at the Institute of Cancer Research and Lawrence Kingsley’s group at the University of Pittsburgh.

Several control non-KS, AIDS-related samples being studied by Anne Matsushima, Cesarman, and Knowles in the initial KS tumor screen, however, were also strongly positive for the KS DNA fragments. These samples belonged to an unusual B cell lymphoma, initially called body cavity-based lymphomas (BCBLs) and later renamed primary effusion lymphomas (PELs) (89). Fortunately, cell lines from these lymphomas had been established and one cell line (BC-1) was thawed, cultured, and found to be highly positive for the KS RDA DNA fragments as well as for EBV coinfection (90). This cell line provided the first cell culture system for growing the putative virus, and we were able to generate a serologic test for the virus by first pre-adsorbing sera with paraformaldehyde-fixed P3HR1 EBV-positive cells to remove cross-reactive antibodies. The BC-1 cells bound KS patient sera antibodies with a speckled, nuclear pattern called latency-associated nuclear antigen (91) or LANA. Among AIDS-KS patients, 13 of 14 were positive for LANA antibodies, whereas only 5 of 16 AIDS patients without KS were similarly positive. Shou-Jiang Gao, the first postdoctoral scientist recruited to our new lab, used the PEL cells to identify the high molecular mass LANA protein bands by immunoblotting (92). He would use this assay on Kingsley’s stored sera from the Multicenter AIDS Cohort Study to establish that KSHV infection precedes the onset of KS disease, a temporal relationship that is central to causation (56).

Most of this early work on KSHV was performed without actually knowing the identity of the agent and was subsequently published after the initial description of KSHV (86). With the help of Frank Lee and Janice Culpepper at the DNAX Research Institute, a lambda phage subclone of KS DNA was isolated and sequenced. The BLAST sequence alignment tool (93) had only recently become widely available, and using it on the cloned sequences showed that they clearly belong to a new herpesvirus similar to, but distinct from, squirrel monkey herpesvirus saimiri and human EBV. As the eighth human herpesvirus it was named Kaposi sarcoma-associated herpesvirus (KSHV or HHV8).

Once the initial findings were published (86), patterns for human infection predicted by Beral et al. could be established using the LANA assay. Dean Kedes and colleagues from Don Ganem’s lab found, for example, that asymptomatic gay and bisexual men have high rates of LANA seropositivity and KSHV infection, consistent with sexual transmission (94), while our group showed elevated infections among Italian and Ugandan blood donors that tracked with disease prevalence (95). Despite initial controversy (96, 97), it quickly became clear with independent findings from multiple groups that KSHV fulfills Hill’s criteria for causing KS (58). Furthermore, the origin of the AIDS-KS epidemic was the result of a collision between two different viruses, HIV and KSHV, in high-risk populations. KSHV is also the likely cause of most cases of multicentric Castleman disease (98) and a cytokine-induced inflammatory syndrome (99).

Identifying only a small unique nucleic acid sequence is sufficient to allow the characterization of an entire virus. The original KSHV DNA fragments identified by RDA comprise less than 1% of the ~165 kbase KSHV genome, but these unique sequences allowed development of diagnostic tests and sequencing of the entire viral genome (100). KSHV is a rhadinovirus related to EBV, a lymphocryptovirus, and the two viruses have homologous structural capsid and replication genes. KSHV nonstructural genes, however, are not conserved with those of EBV and encode readily recognizable immune signaling gene lookalikes that act as oncogenes capable of initiating cell proliferation and transformation (26, 101, 102).

The evolution of this virus tells us some things about the likelihood of finding new human viruses. Old World primates possess two different rhadinovirus lineages related to KSHV (103, 104). Both lineages infect higher primates including chimpanzees and gorillas (105, 106), and the second lineage can cause neoplastic disorders in its primate host (107) but has not been found in humans. Either humans are unique among primates in having lost this viral lineage or the human virus still awaits discovery. Molecular evolutionary studies also show that KSHV was once a near-ubiquitous human infection—like most of the other human herpesviruses—but was selected out from many modern populations around the world (108). This relatively poor adaptation of KSHV to humans compared to the other herpesviruses bolsters optimism for a sterilizing vaccine that can be used in LMICs to prevent KSHV infection (109).

**Figure 3:**
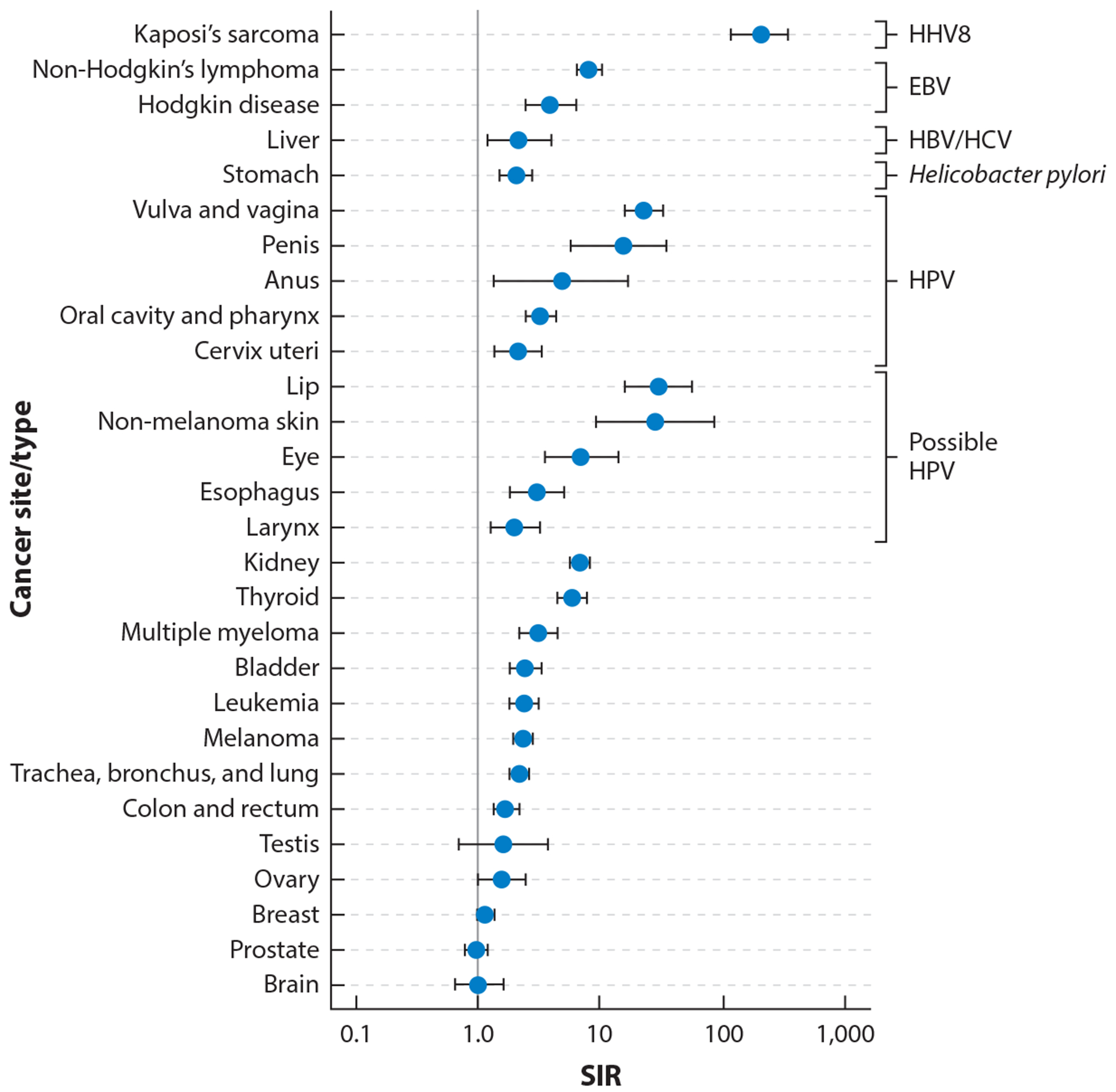
SIRs for site-specific cancers in solid organ transplant recipients showing reliance, as a group, on immune competence in the prevention of known virus-associated cancers. KSHV (HHV8) has a remarkably high SIR as compared with HPV in cervical cancer despite HPV’s obligatory role in cervical cancer. Abbreviations: EBV, Epstein-Barr virus; HBV, hepatitis B virus; HCV, hepatitis C virus; HHV8, human herpesvirus 8 or KSHV, Kaposi sarcoma herpesvirus; HPV, human papillomavirus; SIR, standardized incidence ratio. Figure adapted with permission from Reference 131.

One lesson learned from KSHV is the importance of epidemiology to human cancer virus discovery. KS, a cancer, behaves like an infectious disease, which of course it is as well. KS is more common in immunosuppressed persons and has geographic and risk factor patterns consistent with a communicable carcinogenic agent. Figure 3 shows standardized incidence ratios for various cancers among transplant patients compared to the general population (a similar comparison can be made for AIDS patients). While KS is extreme in its predilection for immunocompromised patients, HPV, EBV, HBV/HCV, and even *Helicobacter pylori* bacteria-related cancers are significantly increased in immunosuppressed populations, as expected for agents expressing foreign antigens. This type of analysis is important for predicting which target cancers should be looked at when searching for infectious etiologies.

Many of the cancers common to Northern Americans and Europeans (e.g., breast, colon, prostate, brain), however, are not significantly increased in immunosuppressed persons. Based on current evidence, these tumors seem unlikely to be good targets to search for new human cancer viruses. Glioblastoma, for example, has been posited to be linked to CMV infection, but it is not significantly increased in immunosuppressed populations nor are CMV transcripts found at significant levels in these neoplasias (110). Based on the known virology of CMV, it is difficult to conceive of a mechanism by which it could cause glioblastomas without provoking an immune response or measurable viral gene expression.

## MERKEL CELL POLYOMAVIRUS

Starting in 1998, we turned our attention to other technologies that might help find cancer viruses. RDA worked for KSHV but is limited in requiring the candidate cancer virus to have a large DNA-based genome. We reasoned that if an infectious agent causes a cancer and the agent is a direct carcinogen (111), it would express an oncoprotein antigen in each and every cell. This suggested that the way to find a cancer virus would be complementary DNA (cDNA) sequencing. Direct cDNA sequencing has another advantage over physical genome subtraction because it simplifies the search to the 2% of the genome that encode mRNAs.

To determine how deep the sequencing would have to be to find a virus, we first needed to know what fraction of the transcriptome, in a virally infected cancer cell, is composed of viral mRNAs. Gene expression studies on monomorphous (uniform cell) virus-infected cell lines (e.g., HeLa) suggested viral mRNAs comprise ~0.1% of the total cellular transcriptome. Assuming a binomial distribution for successfully finding a foreign cDNA at this transcript prevalence, random cDNA sequencing would require sequencing 980 cDNAs (or ten 98-well plates with cloned cDNAs) to achieve an ~60% success rate in finding a single viral cDNA. We first attempted to achieve this using serial analysis of gene expression to concatemerize 3′ end tags of cDNAs as a cost-saving approach as early as 1999 (112), but these early experiments did not become practical until the draft of the human genome was completed in 2001 (51).

Digital transcriptome subtraction (DTS) algorithms were developed to differentiate human from nonhuman sequences (Figure 2), and 240,000 expression tags from Ugandan squamous cell conjunctival carcinoma, a cancer having a suspected viral origin, were examined (113). Although no unique viral mRNAs were identified, this level of sequencing ruled out a novel virus transcript being present in the conjunctival carcinomas examined at the level of 4 transcripts per cell or greater. Meyerson and colleagues’ laboratory (114) had also independently developed a similar sequencing-based approach to identifying new viruses.

In 2002, Engels and colleagues noted the high rate of MCC among AIDS patients compared to the general population (115), a pattern reminiscent of KS. MCC is an aggressive skin cancer; however, it is uncommon (~3,000 cases in the United States each year), and MCC tumor banking was rare. It required several years for us to acquire a handful of cases having intact RNA. Sequencing technology (454 sequencing) also had matured by early 2007 such that hundreds of thousands of sequences could be sequenced for ~$20,000 USD per sample. These sequences were compatible with our DTS algorithms, and the longer read lengths would markedly enhance self-versus nonself-discrimination.

Two cDNA libraries were prepared from 4 dissected MCC tumors to obtain nearly 400,000 high-fidelity sequences. Of these, 99.5% aligned to the human RefSeq database using DTS. The remaining 2,395 sequences formed a candidate pool that was aligned to viral sequence databases. Only one unique cDNA, which was fused to a human receptor tyrosine phosphatase transcript, aligned with a highly conserved DNA binding site on the large T (LT) antigen gene of African green monkey polyomavirus (67). Notably, the viral transcript had the identical fusion in both the primary MCC tumor and metastasis, indicating that this was a clonal insertional mutation (67). Genome walking then allowed us to predict the putative closed circular genome of MCV. MCV has no way to recircularize its integrated genome to form replication-competent viruses, and tumor formation is a dead-end event for the virus. It is now widely recognized that MCV causes 60–80% of MCC cases. The remaining virus-negative MCCs have abundant UV-associated mutations in cellular tumor suppressor pathways producing cancers that phenocopy the effects of an integrated MCV genome (52).

In addition to clonal insertion into the host genome, tumor-derived MCVT antigen genes also carry mutations that were initially perplexing because the T antigen gene seemed certain to be a viral oncogene involved in cell transformation (68). But close examination of these mutations led to interesting insights into the molecular pathogenesis of virus-driven MCC (116). It now seems clear that if the virus genome fragments during replication (117), linear viral genome fragments can be inserted into the host chromosome DNA. If a wild-type LT helicase protein is expressed from an inserted viral DNA, it can bind back onto the now-integrated viral origin, initiating unlicensed DNA replication at the site of the integration. This will cause replication fork collisions and DNA fragmentation that will likely kill the nascent tumor cell. Tumor cells can survive this bottleneck only if the virus genome is integrated (to maintain the viral DNA) and possesses a mutation to LT protein that inactivates its helicase replication activity (68). This results in unopposed expression of the T antigen genes that drive Merkel cell cancer transformation (118). When these two rare molecular events occur in combination, together with loss of cellular immunity against early viral antigens, for example, through AIDS or aging, then this common skin flora virus can turn into an aggressive cancer agent.

## PROSPECTS FOR ADDITIONAL TUMOR VIRUS DISCOVERIES

MCV was the first human pathogen discovered through nondirected transcriptome sequencing. The likelihood for discovery of additional cancer viruses using tumor transcriptome databases seemed certain to occur if such additional cancer viruses exist. As described for the absence of HCV sequences in TCGA, however, this very much depends on how the tumor RNA is sequenced. Most tumor transcriptome studies are focused on coding mRNAs. Some viruses, such as herpes simplex, express only noncoding RNAs during latency (119). Noncoding viral RNAs, including viral microRNAs (120) and circular RNAs (121, 122), are likely to contribute to viral tumorigenesis but require specialized sequencing methods for detection.

Even if a tumor virus does express protein-encoding mRNAs, there is the possibility that the RNA will fail to be found during sequencing. Some latent viral proteins, such as EBV EBNA1 and KSHV LANA1, evade cellular peptide presentation to major histocompatibility complex I by being extremely stable and avoiding formation of defective ribosomal products (123–127). This may be a more common mechanism than is recognized as a means for persistent viral evasion of cellular immunity. Only a scant amount of mRNA is required to generate functional amounts of these superstable proteins, so their mRNAs can easily be missed in tumor transcriptome studies.

We have tried to address this problem by using a proteomic-based analog to DTS that we call differential peptide subtraction (128) (Figure 2). Mass spectrometry can interrogate hundreds of thousands of peptide features and can be applied to archived formalin-fixed tissue specimens with degraded RNA. This is particularly important for rare tumors obtained from pathology tissue banks. In a proof-of-principle experiment surveying nearly 500,000 peptide features obtained from a blinded collection of MCV-positive and MCV-negative Merkel cell carcinomas, we found 20 unique, nonhuman peptide features that were present only in virus-positive MCC and not in virus-negative MCC. Four (20%) of these peptides were derived from the MCV large T protein, showing that a nondirected search for virus peptides is readily achievable (128). Reverse translation of candidate peptides leading to degenerate DNA sequences then can be obtained to allow PCR cloning of larger portions of the viral genome and detection of the virus. This approach might be most useful in the case of rare immune-related cancers, such as EBV-negative post-transplantation lymphoma (114), in which no viral RNAs have been detected but evidence suggests that a viral protein antigen is present.

To some it might be disappointing that the cancer transcriptome revolution has not uncovered additional new cancer viruses. But it is important to be cautious before concluding that there are no more cancer viruses left to be discovered. There are clearly human cancers, particularly cancers important to LMICs, that have not benefited from saturation sequencing studies. Circuloviruses, ERV and other endogenized viruses, are commonly present in cells and would be difficult to investigate as causes of cancer by transcriptome sequencing. Many cancer genomic studies are based on technologies such as hybrid capture that are not likely to identify viral DNA even if present and abundant, unless it is found as an off-target sequence such as occurred for HPV42 and digital papillary adenocarcinoma (129). Also, the genetically heterogeneous nature of cancer means that it is difficult to establish causality in cases where a virus is responsible for only a fraction of the cancers being examined. Finally, even if there are currently no more human tumor viruses left to be discovered, this does not mean that there will be no more cancer viruses in the future. The global SARS-CoV-2 and HIV pandemics reveal the importance of zoonotic viruses, and there is no reason to suppose that a zoonotic viral infection, particularly one that is poorly adapted to replicate in human cells, could not cause a future cancer pandemic. If such a zoonotic cancer infection occurred, it may not be recognized as a cancer risk factor for decades after the initial infection occurred.

Taken together, we do have a definitive answer to the Editorial Committee’s question, “Are there more human cancer viruses left to be found?” The answer is a resounding “Maybe.”

## Figures and Tables

**Table 1:** Viruses associated with human cancers

Year	Virus	Abbreviation	Baltimore classification		Notable cancer(s)
1964	Epstein-Barr virus	EBV	I	dsDNA virus	Burkitt lymphoma, nasopharyngeal carcinoma, Hodgkin disease, gastric cancer
1965	Hepatitis B virus	HBV	VII	dsDNA-RT virus	Hepatocellular carcinoma
1980	Human T-lymphotropic virus 1	HTLV-1	VI	ssRNA-RT (+) sense RNA with DNA intermediate in life cycle	Adult T cell leukemia
1983	Human papillomavirus	HPV	I	dsDNA-RT virus	Cervical and other anogenital cancers, head and neck cancers, digital papillary adenocarcinoma
1989	Hepatitis C virus	HCV	IV	(+) ssRNA virus	Hepatocellular carcinoma
1994	Kaposi sarcoma herpesvirus	KSHV	I	dsDNA virus	Kaposi sarcoma, primary effusion lymphoma, multicentric Castleman disease
2008	Merkel cell polyomavirus	MCV (MCPyV)	I	dsDNA virus	Merkel cell carcinoma
1983	Human immunodeficiency virus^a^	HIV	VI	ssRNA-RT (+) sense RNA with DNA intermediate in life cycle	Spectrum of cancers associated with CD4^+^ immunosuppression
1971	BK polyomavirus^b^	BKV (BKPyV)	I	dsDNA virus	Urothelial carcinoma

a Promotes cancers by other viruses.

b Iatrogenic cancer only.

Abbreviations: dsDNA, double-stranded DNA; RT, reverse transcription; ssRNA, single-stranded RNA. Table adapted from Reference 18 and Reference 132.
